# Gut microbiota and ulcerative colitis: a bibliometric analysis of knowledge structure, research hotspots, and future directions

**DOI:** 10.3389/fmicb.2026.1765748

**Published:** 2026-02-26

**Authors:** Zhen Zhang, Xiangcheng Hu, Yitong Ma

**Affiliations:** 1 Department of General Surgery, Jinhua Central Hospital Pan‘an Branch, Pan'an, Jinhua, Zhejiang, China; 2 Digestive Endoscopy Center, The Second People's Hospital of Lishui, Lishui, Zhejiang, China; 3 Department of General Surgery, The Second People's Hospital of Lishui, Lishui, Zhejiang, China

**Keywords:** fecal microbiota transplantation, gut microbiota, short-chain fatty acids, traditional Chinese medicine, ulcerative colitis

## Abstract

**Background:**

Ulcerative colitis (UC), a globally prevalent immune-mediated colonic disorder, is fundamentally linked to intestinal dysbiosis. Despite the exponential growth in related papers, systematic, data-driven bibliometric analyses including global productivity trends, international collaboration networks, citation impact distributions, and the temporal evolution of research topics remain lacking.

**Methods:**

We conducted a comprehensive bibliometric analysis of 5,879 articles and reviews sourced from the Web of Science Core Collection (WOSCC) and Dimensions (2004–2025). Publication outputs, international collaboration networks, institutional productivity, and keyword evolution were visualized using R-bibliometrix, VOSviewer, and CiteSpace. Lotka's law and Bradford's law were applied to assess author and journal productivity distributions, respectively. Burst detection algorithms identified emerging research frontiers.

**Results:**

Annual publications demonstrated exponential growth, escalating from 36 in 2004 to a projected 819 in 2024. Geographically, China dominated absolute output (*n* = 2,559), followed by the USA (*n* = 1,181), with these two nations collectively accounting for 63.6% of global publications, justifying their prominence as the two major hubs in this research field. Harvard Medical School exhibited the highest citation efficiency (296.6 citations per publication), contrasting with volume leaders like Zhejiang University (92 publications). Co-occurrence clustering revealed 18 distinct knowledge domains, converging on five accelerating frontiers: “fecal microbiota transplantation (FMT),” “short-chain fatty acids,” “traditional Chinese medicine,” “intestinal barrier mechanisms,” and “nanoparticle-based microbiota modulation.” Burst analysis confirmed these themes-initiated citation surges post-2017, with “nanoparticles” and “intestinal barrier” exhibiting the strongest recent momentum (2023–2025), indicating a paradigm shift from descriptive microbiome profiling to mechanistic, precision-targeted interventions.

**Conclusion:**

The UC-microbiome research agenda has transitioned from correlative association studies to multi-layered therapeutic modulation. Future efforts should prioritize standardizing FMT protocols through randomized controlled trials, establishing multi-ethnic longitudinal cohorts to address population-specific microbiome signatures, elucidating dose–response relationships of microbial metabolites, and converging nanodelivery systems with microbiome engineering to optimize therapeutic precision and sustain remission.

## Introduction

Ulcerative colitis (UC) is an immune-mediated chronic inflammation of the large intestine characterized by recurrent episodes of inflammation and remission in the colonic mucosa (Iyengar et al., 2024). Entering the 21st century, the incidence of UC has rapidly increased in newly industrialized countries, particularly in China. This has made UC a truly global disease, imposing a significant economic and disease burden on society and affected families (Wan et al., 2024). Patients often experience debilitating symptoms, including abdominal pain, diarrhea, rectal bleeding, tenesmus, weight loss, extraintestinal manifestations, and an increased risk of colorectal cancer (Du and Ha, 2020). The complex pathophysiology of UC is attributed to the combined effects of genetic and environmental factors, leading to immune dysregulation. This includes impaired mucin synthesis, increased activation of inflammatory cytokines, imbalance of regulatory and effector T cells, and dysbiosis of the gut microbiota (Algieri et al., 2015). The current treatment for UC primarily involves anti-inflammatory agents and immunosuppressants, such as aminosalicylates, corticosteroids, immunosuppressive drugs, and biologic therapies. However, these approaches are often associated with infections, fever, diarrhea, and high recurrence rates (Chen J. et al., 2023; Chen L. A. et al., 2023; Gros and Kaplan, 2023). Consequently, the treatment of UC has become a highly researched field, with an urgent need to identify safe, cost-effective, and efficient therapeutic options.

In recent years, multiple studies have demonstrated that gut microbiota imbalance (i.e., dysbiosis) plays a significant role in the pathogenesis of UC (Zhao et al., 2022; Carlson et al., 2023; Gong et al., 2024). UC patients exhibit significantly reduced intestinal microbial diversity, with decreased levels of beneficial bacteria (e.g., Bifidobacterium, Lactobacillus) and relative increases in harmful bacteria (e.g., certain Bacteroides species, Enterobacteriaceae). This imbalance may disrupt intestinal barrier function and promote inflammatory responses (Shi et al., 2023; Zhao et al., 2024). Dysbiosis leads to abnormal immune recognition, activates inflammatory pathways, and fuels the inflammatory process in UC (Qin et al., 2024). Microbiota-modulating therapies, including probiotics, fecal microbiota transplantation (FMT), dietary adjustments, and traditional Chinese medicine (TCM), have demonstrated potential in alleviating UC symptoms, underscoring the critical role of gut microbiota in UC (Hu et al., 2024; Ma et al., 2024). Therefore, intestinal dysbiosis is a significant factor in the onset and progression of UC. Modulating microbial balance holds broad prospects for UC treatment, making it crucial to analyze the current research status and development trends in this field.

As early as 1969, Pritchard proposed the concept of bibliometrics, which is defined as “the application of mathematical and statistical methods to the calculation and analysis of different aspects of text information to reveal the process of text information and the nature and trend of discipline development” (Zhang et al., 2024). In recent years, bibliometrics has been widely used to explore the characteristics of academic publications in specific fields of research: influential countries, journals, institutions, and authors; Favorable publications, references and keywords (Ma et al., 2021). Bibliometrics is a discipline that employs mathematical and statistical methods to conduct quantitative analysis of the quantity, structure, and patterns of change in scholarly materials such as academic papers, patents, and books. Through statistical analysis of bibliographic data—including publication counts, citation frequencies, and author collaborations—it reveals the patterns governing the production, dissemination, and utilization of scientific literature. This facilitates understanding of disciplinary trends, research hotspots, and scientific collaboration networks (Ninkov et al., 2021).

Despite the rapid expansion of research linking gut microbiota to UC pathogenesis, a systematic, quantitative characterization of the underlying knowledge architecture remains elusive. Specifically, the evolutionary trajectory of research themes, the structural patterns of global scientific collaboration, and the temporal dynamics of emerging frontiers have not been rigorously delineated.

To bridge these gaps, this study employs bibliometric mapping to address four specific research questions: (i) How has the publication volume and productivity distribution among authors evolved temporally (2004–2025), and does it conform to established bibliometric laws (i.e., Lotka's and Bradford's laws)? (ii) What are the structural characteristics of international and institutional collaboration networks, and which entities serve as pivotal nodes in knowledge dissemination? (iii) How can the vast research corpus be systematically categorized into thematic clusters? (iv) Which specific research frontiers are currently experiencing citation bursts, thereby indicating nascent trends in the transition from mechanistic understanding to clinical translation?

This study employs quantitative analysis to reveal the academic landscape in the field of UC-microbiome research, identify knowledge gaps between basic research and clinical applications, and provide evidence-based guidance for prioritizing future research directions.

## Material and methods

### Database and search strategy

We selected the Web of Science Core Collection (WOSCC) and Dimensions databases for literature retrieval. For WOSCC, we searched the Science Citation Index Expanded (SCI-Expanded) and Social Sciences Citation Index (SSCI) using the advanced search interface. The search query was constructed as follows: TS = (“Colitis, Ulcerative” OR “Idiopathic Proctocolitis” OR “Ulcerative Colitis” OR “Colitis Gravis” OR “Inflammatory Bowel Disease, Ulcerative Colitis Type”) AND (“gut microflora” OR “gut microbiota” OR “gastrointestinal flora” OR “gut flora” OR “gastrointestinal microbiota” OR “gut microbiome” OR “gastrointestinal microflora” OR “intestinal microbiome” OR “intestinal microbiota” OR “intestinal microflora” OR “intestinal flora” OR “enteric bacteria”). The search fields included Title, Abstract, Author Keywords, and Keywords Plus. Publication type was restricted to “Article” or “Review,” and language to “English.”

For Dimensions, we utilized the “Full Data” search mode with the following query structure: (“Colitis, Ulcerative” OR “Idiopathic Proctocolitis” OR “Ulcerative Colitis” OR “Colitis Gravis” OR “Inflammatory Bowel Disease, Ulcerative Colitis Type”) AND (“gut microflora” OR “gut microbiota” OR “gastrointestinal flora” OR “gut flora” OR “gastrointestinal microbiota” OR “gut microbiome” OR “gastrointestinal microflora” OR “intestinal microbiome” OR “intestinal microbiota” OR “intestinal microflora” OR “intestinal flora” OR “enteric bacteria”). Given Dimensions' broader indexing of preprints and conference proceedings, we applied additional filters to restrict results to peer-reviewed journal articles and reviews published in English. The time span for both databases was restricted from January 1, 2004, to October 24, 2025.

After finalizing the dataset of literature to be analyzed, metadata and citation data were systematically extracted. Leveraging Web of Science's robust citation network analysis capabilities, citation metrics [total citations and average citations per paper (CPP)] primarily referenced the Web of Science Core Collection. For the minimal number of publications not indexed in Web of Science, citation metrics from the Dimensions database were consulted to ensure maximum consistency and prevent double-counting bias. No attempt was made to merge citation counts from both databases, as this would introduce inflationary bias due to differing citation indexing policies and temporal coverage between WOSCC and Dimensions.

Specifically, for each publication, we extracted the “Times Cited” count from WOSCC as of October 24, 2025. For institutional and national citation analyses, we calculated:

Total Citations: Sum of all WOSCC citations received by publications affiliated with a specific institution or country.

Average Citations per Paper (CPP): Total Citations divided by the number of publications (*N*) from that entity, i.e., CPP = (∑Citations)/*N*.

### Eligibility criteria and screening protocol

**Figure 1:**
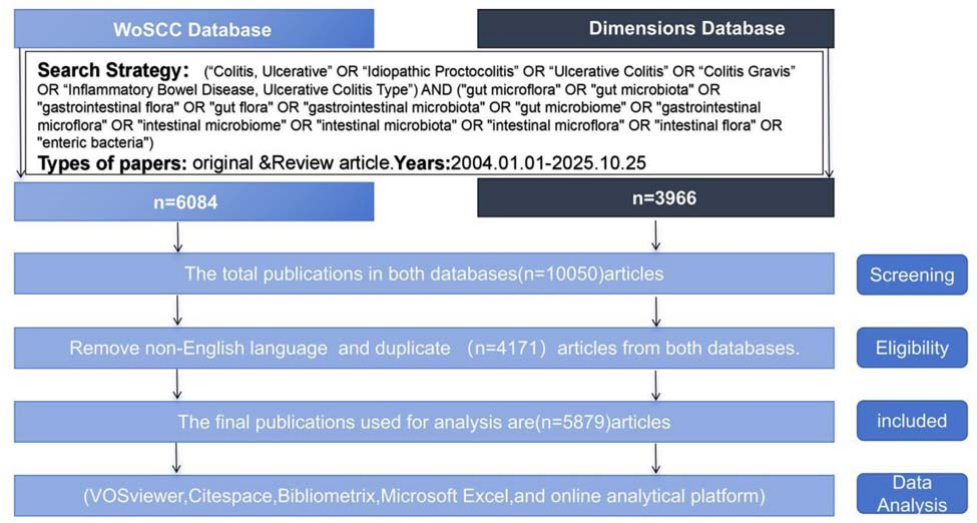
PRISMA flowchart for screening and retrieval.

The literature screening process strictly adhered to the Preferred Reporting Items for Systematic Reviews and Meta-Analyses (PRISMA) guidelines, with the workflow illustrated in Figure 1. **Screening:** Initially, we retrieved 10,050 records from WOSCC and Dimensions databases through the aforementioned search strategy. **Eligibility**: All retrieved records were imported into EndNote 20 software, where the duplicate detection features automatically identified and removed 4,171 duplicate entries by comparing titles, authors, journal names, publication years, languages, and DOIs. **Included**: Ultimately, the two independent researchers screened the titles and abstracts of 5,879 records. Records were excluded if they were: (i) irrelevant to ulcerative colitis or gut microbiota; (ii) editorials, letters, meeting abstracts, or book chapters; (iii) written in non-English languages; or (iii) published outside the 2004–2025 timeframe. Following full-text review, all 5,879 articles met the inclusion criteria and were retained for bibliometric analysis. Any disagreements during the screening process were resolved through discussion or consultation with the corresponding author until consensus was achieved.

### Data analysis

We created flow charts in Microsoft word 2019 and statistical tables and fitted curve analyses in Microsoft Excel 2019. Lotka's law analysis and Bradford's law analysis were performed using bibliometrix 4.1.3 tool in R 4.3.1 software. Through the online bibliometrics website (https://bibliometric.com/), we were able to visualize international collaborations between countries and Origin 2024 was utilized to perform national visualization analysis. In addition, VOSviewer 1.6.19 and Citespace 6.2R4 were used for bibliometric analysis of institutions, authors, journals, keyword. The main focus was to examine co-authorship, co-occurrence, and co-citation patterns. We combined overlapping items into a unified element, corrected misspelled words by manual means, and performed data cleaning before exporting the data for subsequent analysis.

### Lotka's law and Bradford's law

Lotka's law, based on a power-law distribution, describes the relationship between an author's productivity and his publication frequency (Sharma and Chariar, 2024). It states that the number of authors who have published a single article is significantly higher than the number of authors who have published multiple articles. Specifically, Lotka's law was employed to quantify the inequality in author productivity—revealing whether the field is dominated by a small elite of highly productive researchers or characterized by broad, decentralized participation. This concentration index provides insight into the field's intellectual maturity and the stability of its knowledge base. Lotka's law is expressed in mathematical terms by the following formula (Ahmad et al., 2022):


$$\begin{array}{l} A\left(n\right)=A\left(1\right)/n^{2} \end{array}$$

In the above equation, *A*(*n*) is the number of authors publishing *n* papers and *A*(1) is the number of authors publishing a single paper.

Author productivity was assessed by extracting publication counts for all standardized author names, constructing a frequency distribution of authors by their output level (*n* papers), and fitting the data to the power-law model *A*(*n*) = *C*/*n*∧α using logarithmic transformation (ln[*A*(*n*)] = ln*C* – αln*n*) followed by ordinary least squares regression to estimate the exponent α and constant *C*; model conformity was validated via coefficient of determination (*R*^2^) and Kolmogorov-Smirnov testing, supplemented by Gini coefficient calculation and core author identification.

Bradford's law was applied to identify the core journal set that concentrates the majority of publications, thereby distinguishing essential literature sources from peripheral scattering. Together, these bibliometric distributions enable us to assess the hierarchical structure of knowledge production—indicating whether the field is centralized around high-impact hubs or fragmented across dispersed sources—critical information for researchers seeking to navigate the literature efficiently and identify authoritative voices. In this sense, Journals were ranked by descending publication count and partitioned into three zones using the Leimkuhler model: the core zone (*r*0 = *k*·ln*k* journals), related zone (*r*0·*k* journals), and scatter zone (*r*0·*k*^2^ journals)—where *k* represents the Bradford multiplier derived from the least productive journal's article count, with the characteristic *S*-shaped distribution verified via Bradford plots (cumulative articles vs. logarithm of cumulative journals; da Silveira Guedes, 2012).

## Result

We retrieved a total of 10,050 published papers from the WOSCC and Dimensions databases. Based on selection criteria including publication date, article type, and language, 5,879 papers were ultimately selected for bibliometric analysis. The specific workflow is illustrated in Figure 1.

### Analysis of the number of publications

**Figure 2:**
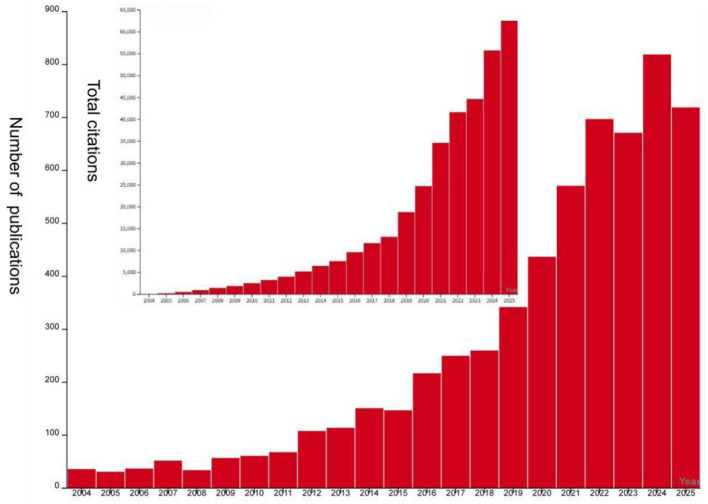
Trend chart of annual growth and citation trends of literature related to gut microbiota and UC.

Figure 2 illustrates the bibliometric trends in UC and gut microbiota research from 2004 to 2025. The number of publications has grown steadily from 36 in 2004 to a projected 819 in 2024, reflecting significant advancement and heightened research interest in this field. Concurrently, the total number of citations has risen from a low baseline in 2004 to 819 citations projected for 2024, reflecting the sustained growth in the academic influence and recognition of research outcomes within this field. Although the projected data for 2025 indicates a slight decline in both publication volume and total citations, the overall figures remain at a high level, suggesting the field will maintain an active research trajectory in the coming years. These data provide crucial bibliometric evidence for understanding the research dynamics and academic impact within this domain.

### Situation of countries/regions and institutions

**Figure 3:**
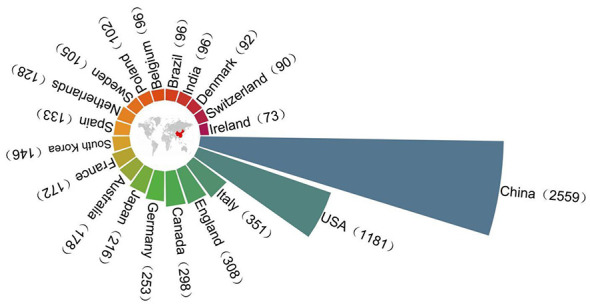
Nightingale's Rose chart of the top 20 countries by publications.

In the field of UC and intestinal microbiome research, Figure 3 reveals significant disparities in the number of published papers across countries. China leads with 2,559 papers, securing its position as a frontrunner in this domain. The United States follows closely in second place with 1,181 papers, demonstrating sustained growth in research activity and outcomes in recent years and gradually establishing its dominance in this field. Additionally, countries such as Italy, the United Kingdom, Canada, Germany, and Japan have also published substantial numbers of papers, each exceeding 200. This indicates that these nations possess a solid research foundation and significant influence in this field. Although countries like Poland, Belgium, Brazil, and India have published relatively fewer papers, they are still conducting foundational explorations and cutting-edge research in this area.

**Figure 4:**
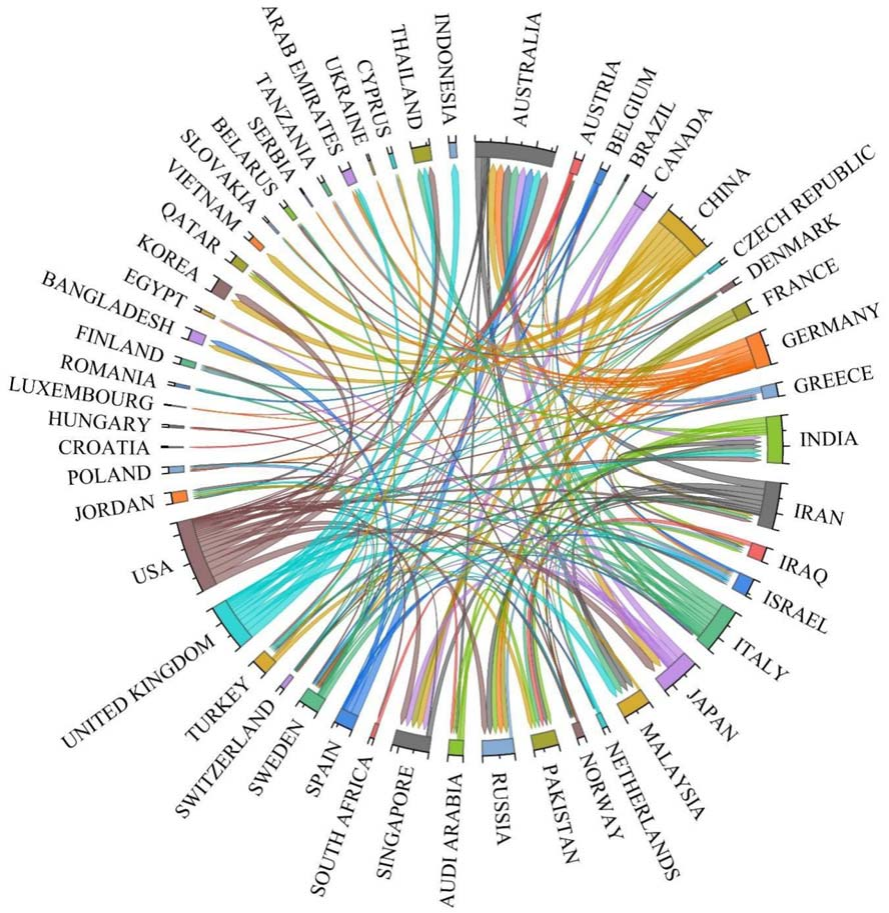
Network of cooperation between countries.

In this study, Figure 4 illustrates the collaborative landscape among countries in the field of gut microbiota research related to UC. As depicted, the central rings in the chord diagram represent participating nations, while the connecting lines between rings denote collaborative relationships. The thickness of these lines reflects the intensity of collaboration, specifically the number of jointly published papers. The figure reveals that the USA has established extensive collaborative networks with multiple countries in this research domain, including China, Australia, and the UK. The USA exhibits the highest number of collaborative connections with other nations, characterized by notably thicker lines, indicating its high level of collaborative activity and influence within this field. China, as an emerging research power, maintains close collaborations with the United States, Japan, Australia, and others. This reflects China's rapid advancement in this field and its potential for international cooperation.

**Figure 5:**
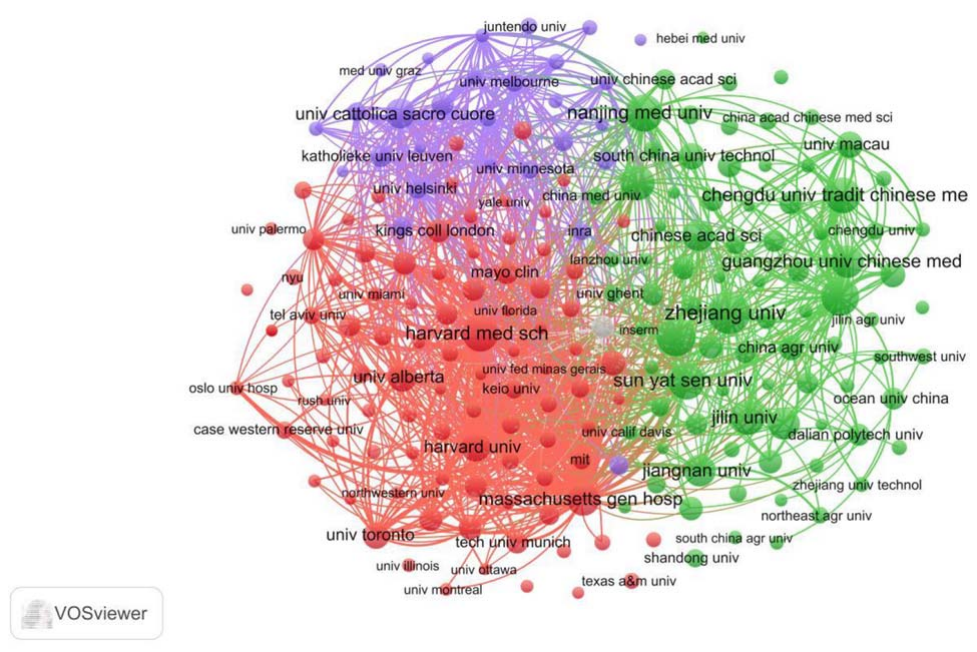
A network visualization map of institutions in the field of gut microbiota and UC. Nodes represent individual institutions, with node size proportional to the total number of published documents (publication volume), and node color indicating distinct clusters. Lines (edges) between nodes represent active collaborative relationships, with line thickness indicating the strength of collaboration (quantified as the total number of co-authored publications between two institutions). Institutions within the same cluster share similar collaboration patterns. The spatial distribution (distance between nodes) approximates the relatedness of institutions based on co-authorship patterns, with shorter distances indicating closer collaborative ties.

The institutional collaboration visualization network diagram (Figure 5), generated using VOSviewer software, clearly illustrates the cooperative relationships among institutions within the field of gut microbiota and UC research. The diagram reveals multiple collaborative nodes within the network, with node size and color intensity reflecting each institution's publication output and collaborative strength. Institutions connected by lines indicate active collaborative research projects, which facilitate knowledge exchange and sharing, thereby advancing progress in this field. The network structure reveals several core institutions positioned centrally, linked to multiple others, playing pivotal roles in leadership and coordination. These core institutions typically possess high research standards and abundant resources, attracting collaboration from other entities. For instance, Zhejiang University may serve as a vital core node in the diagram. Its leading publication volume indicates substantial research output in this field, while its extensive collaborative relationships with numerous institutions provide crucial support for the construction of the entire research network. Internationally renowned institutions like Harvard University and Harvard Medical School also occupy prominent positions in the diagram. Their extensive collaborations with domestic and international institutions introduce cutting-edge research concepts and technologies to the field, elevating its international standards and research quality.

Additionally, the network diagram reveals smaller clusters where institutions collaborate closely, potentially representing distinct research directions or teams. Interconnections between these clusters reflect crossovers and integration among different research approaches, fostering multidisciplinary research development in the field. For instance, institutions primarily focused on TCM research, such as Nanjing University of Chinese Medicine and Guangzhou University of Chinese Medicine, may form a relatively independent cluster. They bring unique perspectives and methodologies to exploring the relationship between TCM and the gut microbiota in UC. This complements the research conducted by institutions primarily engaged in modern medical studies, collectively driving deeper advancements in this field.

According to Table 1, Zhejiang University leads the field of gut microbiota and UC research with 92 publications, totaling 4,293 citations and an average of 46.67 citations per paper, indicating significant academic influence. Shanghai Jiao Tong University and Nanjing University of Chinese Medicine rank second and third with 83 and 78 publications respectively. Both institutions demonstrate active research engagement in this field and have made significant contributions to its advancement.

**Table 1:** The top 20 most prolific organizations in the field of gut microbiota and UC.

**Rank**	**Institution title**	**Documents**	**Total citations**	**Average citation**
1	Zhejiang University	92	4,293	46.67
2	Shanghai Jiao Tong University	83	3,168	38.17
3	Nanjing University of Chinese Medicine	78	2,832	36.31
4	Nanjing Medical University	77	2,897	37.62
5	Sun Yat Sen University	76	2,255	29.67
6	Harvard Medical School	75	9,945	132.6
7	Chengdu University Traditional Chinese Medical	71	2,265	31.9
8	Jilin University	70	1,440	20.57
9	Southern Medical University	70	2,300	32.86
10	Guangzhou University of Chinese Medicine	63	1,859	29.51
11	Massachusetts General Hospital	63	14,670	232.86
12	Jiangnan University	62	1,767	28.5
13	Nanchang University	62	1,741	28.08
14	Chinese Academic Science	58	1,934	33.34
15	Harvard University	56	16,611	296.63
16	Università Cattolica Del Sacro Cuore	56	3,980	71.07
17	University of Alberta	55	4,074	74.07
18	University of Macau	47	1,626	34.6
19	University of Toronto	47	3,798	80.81
20	South China University of Technology	46	1,418	30.83

Institutional distribution reveals that Chinese universities and research institutions dominate the top 20 rankings, underscoring China's robust research capabilities and enthusiasm in gut microbiota and UC studies. Their findings have not only generated significant domestic impact but are also gaining international recognition. For instance, institutions like Nanjing Medical University and Southern Medical University have achieved notable accomplishments in this field, with both publication volume and citation counts ranking highly, reflecting the depth and breadth of China's research in this domain.

Notably, internationally renowned institutions like Harvard Medical School and Harvard University also rank among the top 20 in publication volume. Moreover, their average citations per paper significantly exceed those of other institutions—Harvard Medical School averages 132.6 citations per paper, while Harvard University averages 296.63 citations per paper. This indicates that these international institutions are at the forefront of research in this field, with their findings possessing high academic value and innovation. They play a crucial role in guiding global research directions and development trends in this area.

### Authors analysis

**Figure 6:**
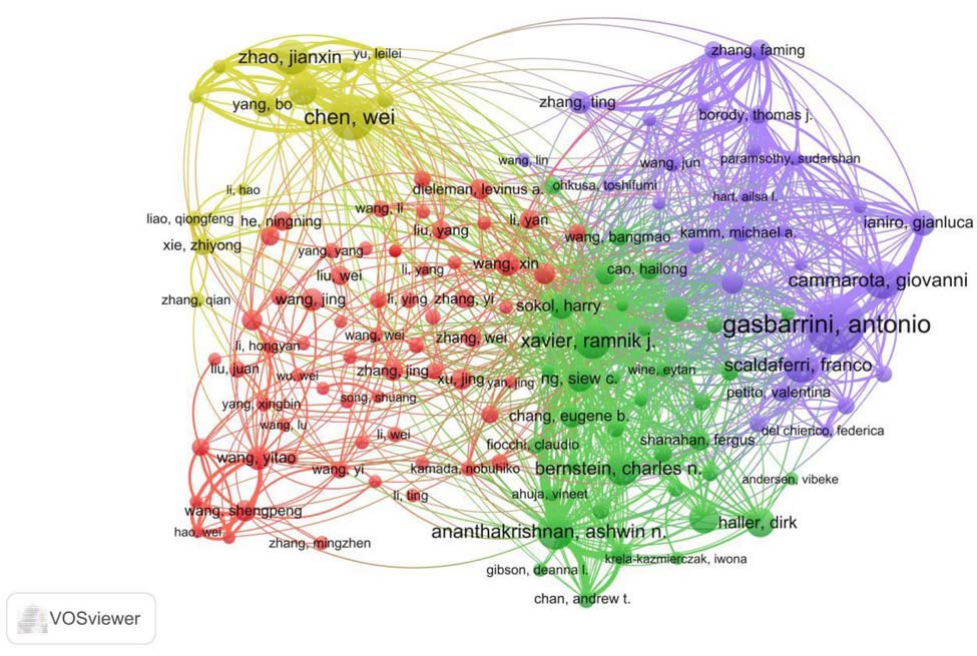
Network diagram of author collaborations for gut microbiota and UC. Nodes represent individual authors, with node size proportional to the author's publication output (number of documents), and node color denoting membership in distinct collaboration clusters. Lines connecting nodes indicate active co-authorship relationships, with line thickness representing the strength of collaboration (number of joint publications). The thickness of the connecting line between two authors correlates with their collaborative frequency; thicker lines indicate more intensive collaboration. Clusters are color-coded to distinguish different research groups or collaborative teams.

This study utilized VOSviewer software to construct an author collaboration network diagram (Figure 6). The diagram illustrates collaborative relationships among authors within this field. Node size represents an author's output volume, while connecting lines denote collaborative ties. The diagram reveals a pronounced collaborative trend in this research domain, with particularly close cooperation observed among certain authors. For instance, the thickest connection links Gasbarrini, Antonio and Cammarota, Giovanni, indicating their strongest collaboration. Additionally, Chen, Wei; Xavier, Ramnik J.; Ananthakrishnan, Ashwin N.; and Wang, Yitao demonstrate robust collaborative ties within their respective clusters. These tight collaborative networks likely represent core research teams within the field, whose cooperation plays a crucial role in advancing its research progress. Furthermore, the network diagram reveals several smaller collaborative clusters, potentially representing secondary research teams or emerging partnerships within the field. The collaboration among these teams also contributes positively to the accumulation of knowledge and academic development in this domain.

**Figure 7:**
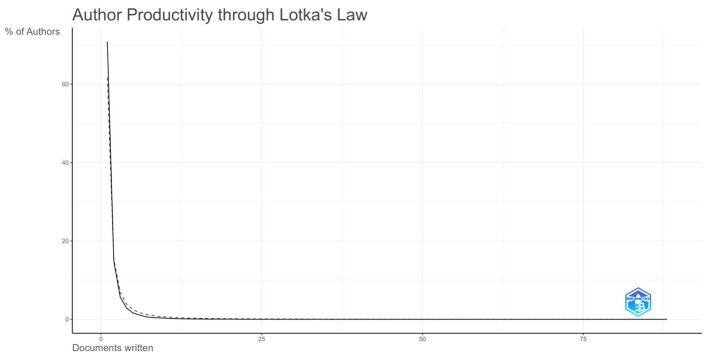
Scientific productivity of authors based on Lotka's law.

The author scientific output diagram based on Lotka's Law (Figure 7) illustrates the distribution of literature output among authors in this field. The figure shows that as the number of publications per author increases, the total number of authors decreases rapidly, consistent with Lotka's Law predictions. This indicates that within the research domain of gut microbiota and UC, a few highly productive authors exist whose research contributions play a pivotal role in advancing knowledge accumulation and academic development within the field. Such concentration suggests the existence of established research leadership and specialized expertise centers, but also highlights potential knowledge gatekeeping effects where emerging researchers face barriers to visibility. The Gini coefficient derived from this analysis further quantified the degree of inequality, providing an objective metric for comparing collaborative openness across different biomedical subfields.

Table 2 lists the top 20 most prolific authors and their citation metrics. As shown in the table, Gasbarrini, Antonio leads with 48 publications and 4,220 total citations, demonstrating his significant influence in the field. Following closely are Chen, Wei and Cammarota, Giovanni, whose publication counts and citation figures also underscore their important standing in the field. Furthermore, Xavier, Ramnik J. holds the highest average citation count at 408.69, potentially reflecting the high impact and recognition of his research contributions.

**Table 2:** The top 20 most prolific and cited authors in the field of gut microbiota and UC.

**Rank**	**Authors**	**Documents**	**Total citations**	**Average citation**
1	Gasbarrini, Antonio	48	4,220	87.92
2	Chen, Wei	37	1,142	30.86
3	Cammarota, Giovanni	30	2,264	75.47
4	Ananthakrishnan, Ashwin N.	29	5,320	183.45
5	Xavier, Ramnik J.	29	11,852	408.69
6	Scaldaferri, Franco	28	1,351	48.25
7	Zhao, Jianxin	28	816	29.14
8	Bernstein, Charles N.	27	3,116	115.41
9	Rogler, Gerhard	24	1,458	60.75
10	Haller, Dirk	23	1,253	54.48
11	Zhang, Hao	23	860	37.39
12	Ng, Siew C.	21	3,417	162.71
13	Sokol, Harry	21	5,283	251.57
14	Ianiro, Gianluca	20	2,152	107.6
15	Colombel, Jean-Frederic	19	3,758	197.79
16	Wang, Xin	19	703	37
17	Wang, Yitao	19	1,013	53.32
18	Yang, Bo	19	492	25.89
19	Chang, Eugene B.	18	1,772	98.44
20	Vermeire, Severine	18	1,517	84.28

### Journals analysis

**Figure 8:**
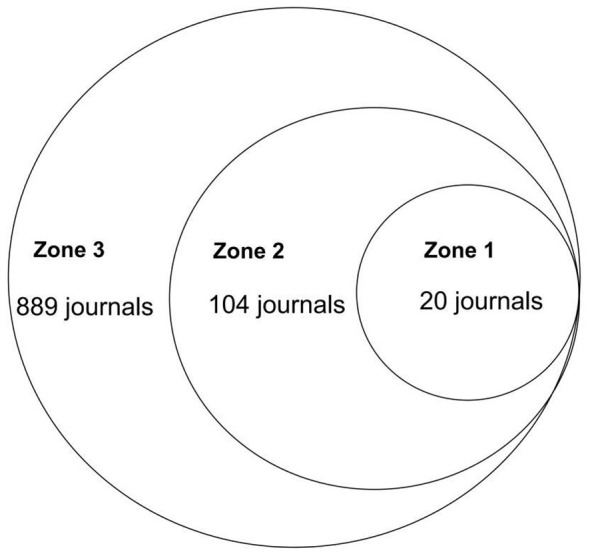
Scientific productivity of journals based on Bradford's law.

Based on Bradford's Law, literature data related to gut microbiota and UC were collected to construct a scientific output map of journals (Figure 8). This map illustrates the volume of literature output across different journals in this research field. It reveals a pronounced pattern of concentration and dispersion, consistent with Bradford's Law distribution: a small number of core journals account for the majority of publications, while the vast majority of other journals contribute relatively few papers. This scattering pattern confirms that while the field draws from a broad disciplinary base (1,013 total journals), information flow is concentrated in high-impact gastroenterology, immunology, and molecular biology venues. For practitioners, this finding has practical utility: it streamlines literature monitoring to a manageable core journal set and identifies optimal publication venues for maximizing visibility within the field.

**Figure 9:**
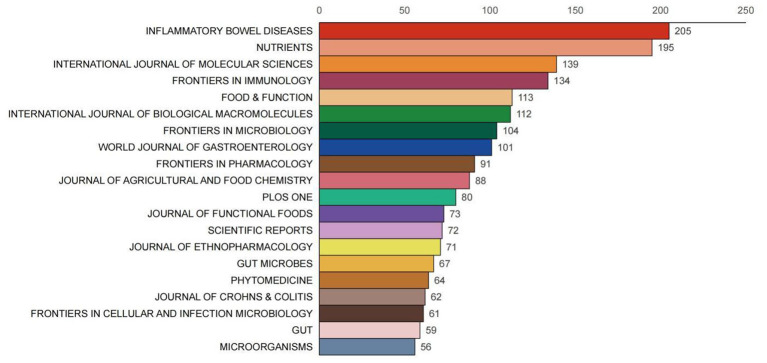
Distribution map of journal publications in zone one.

Based on the scientific output map, journals were further categorized into distinct zones, with Zone 1 journals listed in Figure 9. Ranking Zone 1 journals by publication volume reveals that top-ranked journals not only lead in literature quantity but also exhibit higher citation counts and average citation rates. For instance, INFLAMMATORY BOWEL DISEASES has an average citation rate of 68.55, while NUTRIENTS averages 46.19 citations per article. This further indicates that the research published in these journals possesses high quality and academic value. Concurrently, these journals also boast high impact factors, such as GUT with an impact factor of 25.8 and GUT MICROBES with an impact factor of 11, demonstrating their strong reputation and influence within the field of gastroenterology.

**Figure 10:**
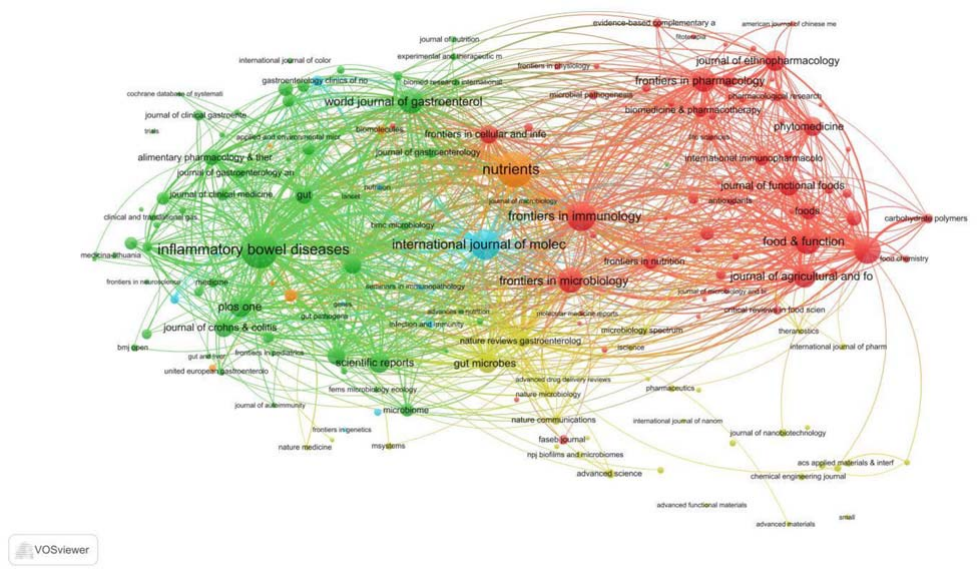
The network diagram of cited journals. Nodes represent individual journals, with node size proportional to the frequency of being cited (citation count), and node color indicating different clusters. Lines between nodes represent co-citation relationships (journals cited together in the same reference list), with line thickness indicating the strength of the co-citation link (frequency of co-occurrence in citing papers). Journals that are frequently co-cited appear closer together in the spatial layout, indicating higher thematic similarity.

Using VOSviewer software, we analyzed collaboration patterns among journals cited in this research field by treating them as nodes, generating a collaborative visualization network diagram (Figure 10). This diagram provides an intuitive overview of collaborative relationships between different journals, revealing which journals exhibit higher citation frequencies and collaboration levels. This approach unveils the research network structure and knowledge dissemination pathways within the field. The figure shows that some journals exhibit frequent citations and collaborations, forming tightly knit network structures. For instance, journals such as FRONTIERS IN IMMUNOLOGY, FRONTIERS IN MICROBIOLOGY, and FRONTIERS IN PHARMACOLOGY likely engage in substantial cross-disciplinary research and collaboration. This indicates significant interdisciplinary integration among immunology, microbiology, and pharmacology in studies concerning gut microbiota and UC. Meanwhile, certain core journals occupy pivotal positions within the network, playing a crucial role in advancing knowledge dissemination and research development across the entire field.

Using Excel software, we collected and organized data on the publication volume, citation counts, average citation counts, and impact factors of journals in Zone 1 (Table 3). These metrics serve as crucial indicators for measuring a journal's influence and academic value. By analyzing this data, we can more comprehensively evaluate the standing and role of each journal within this research field. For example, INFLAMMATORY BOWEL DISEASES, while not the highest in publication volume, exhibits a high average citation count and impact factor, indicating that the research published in this journal possesses high quality and influence. Meanwhile, journals such as NUTRIENTS and INTERNATIONAL JOURNAL OF MOLECULAR SCIENCES hold advantages in publication volume and citation frequency, playing a significant role in advancing research development within this field.

**Table 3:** Leading journals in zone one for gut microbiota and UC studies ranked by issuance and citations.

**Rank**	**Journal**	**Documents**	**Total citations**	**Average citation**	**IF (2025)**
1	INFLAMMATORY BOWEL DISEASES	205	14,053	68.55	4.3
2	NUTRIENTS	195	9,007	46.19	5
3	INTERNATIONAL JOURNAL OF MOLECULAR SCIENCES	139	4,343	31.24	4.9
4	FRONTIERS IN IMMUNOLOGY	134	10,664	79.58	5.9
5	FOOD & FUNCTION	113	3,528	31.22	5.4
6	INTERNATIONAL JOURNAL OF BIOLOGICAL MACROMOLECULES	112	2,491	22.24	8.5
7	FRONTIERS IN MICROBIOLOGY	104	4,666	44.87	4.5
8	WORLD JOURNAL OF GASTROENTEROLOGY	101	7,910	78.32	5.4
9	FRONTIERS IN PHARMACOLOGY	91	2,349	25.81	4.8
10	JOURNAL OF AGRICULTURAL AND FOOD CHEMISTRY	88	2,388	27.14	6.2
11	PLOS ONE	80	5,735	71.69	2.6
12	JOURNAL OF FUNCTIONAL FOODS	73	1,290	17.67	4
13	SCIENTIFIC REPORTS	72	2,763	38.38	3.9
14	JOURNAL OF ETHNOPHARMACOLOGY	71	2,072	29.18	5.4
15	GUT MICROBES	67	4,213	62.88	11
16	PHYTOMEDICINE	64	1,251	19.55	8.3
17	JOURNAL OF CROHNS & COLITIS	62	3,076	49.61	8.7
18	FRONTIERS IN CELLULAR AND INFECTION MICROBIOLOGY	61	2,710	44.43	4.8
19	GUT	59	12,005	203.47	25.8
20	MICROORGANISMS	56	1,061	18.95	4.2

### Keywords co-occurrence, clusters and bursts

This study utilized CiteSpace software to analyze literature on gut microbiota and UC published in the Web of Science database from 2004 to 2025. By constructing keyword co-occurrence maps, clustering diagrams, timeline charts, and emergence maps, it reveals research hotspots, development trends, and key themes within this field.

**Figure 11:**
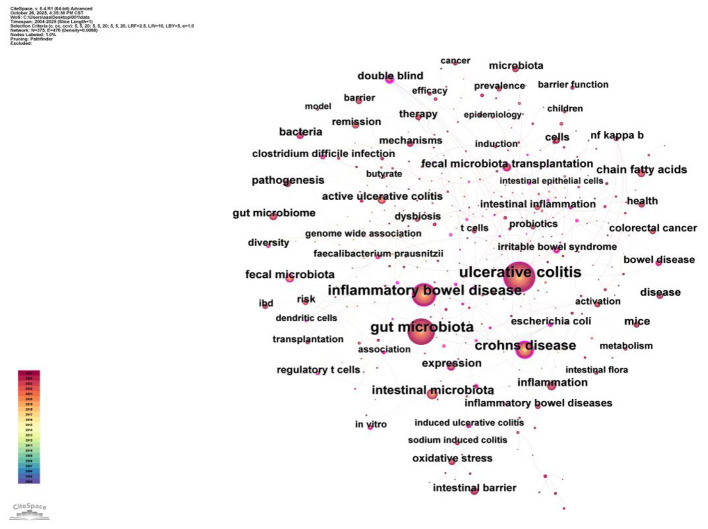
Network map of keywords for gut microbiota and UC studies. Nodes represent keywords, with node size indicating the frequency of keyword appearance (term frequency), and node color representing different clusters. Lines between nodes represent co-occurrence relationships within the same publication, with line thickness and gray-scale intensity indicating the strength of co-occurrence frequency (thicker/darker lines = higher co-occurrence). The purple outer ring on certain nodes indicates high betweenness centrality (keywords acting as bridges between different research areas).

The keyword co-occurrence map (Figure 11) visually displays co-occurrence relationships between keywords, revealing the knowledge structure of the research domain. Node size represents keyword frequency, while connections indicate co-occurrence relationships. The keywords “ulcerative colitis,” “gut microbiota,” and “Crohn's disease” emerge as core research themes, depicted as larger nodes indicating extensive discussion in the literature. Additionally, keywords such as “inflammatory bowel disease,” “intestinal barrier,” and “fecal microbiota transplantation” exhibit high co-occurrence frequencies, reflecting their significant importance in research.

**Figure 12:**
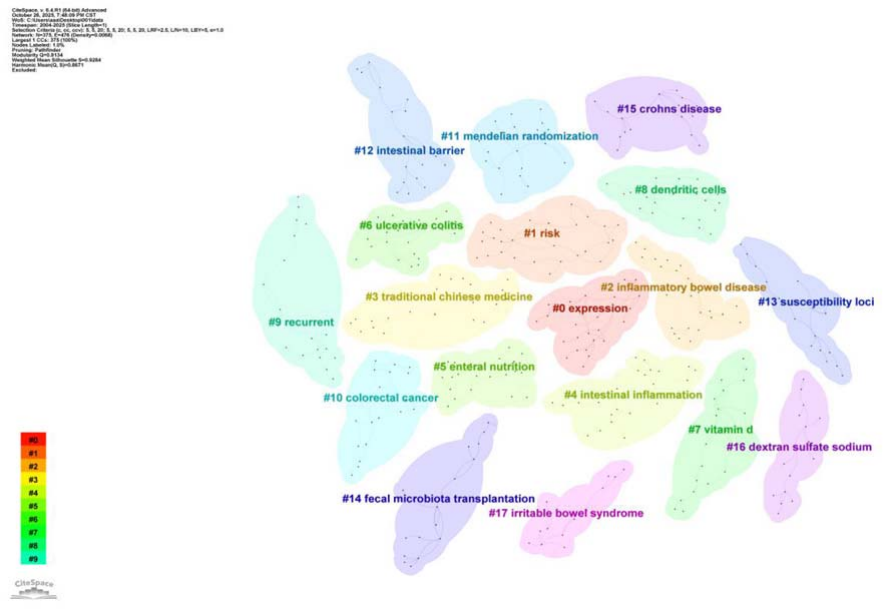
Cluster analysis map of keywords on gut microbiota and UC. Nodes represent keywords positioned horizontally according to their first appearance year and vertically within their respective cluster lanes. Node size reflects keyword frequency, while node color (from cool to warm colors) indicates the temporal progression of research (blue = earlier, red = more recent). Horizontal lines represent cluster time spans, with line thickness indicating the number of containing keywords or citation burst intensity. Cluster identifiers (#0, #1, etc.) are ordered by size, with larger clusters containing more keywords.

The cluster diagram (Figure 12) reveals the thematic structure of the research field by grouping keywords into distinct clusters. Each cluster represents a research theme, with cluster size and color indicating the theme's research activity and duration. The diagram displays 18 major clusters, among which “#0 expression,” “#1 risk,” and “#2 inflammatory bowel disease” are the three largest clusters, indicating these are the primary research themes in this field. Notably, the “#6 ulcerative colitis” cluster aligns with the co-occurrence network results, further validating UC's research prominence in this domain. Additionally, clusters like “#3 traditional Chinese medicine” and “#5 enteral nutrition” highlight the research value of traditional medicine and nutrition in UC treatment.

**Figure 13:**
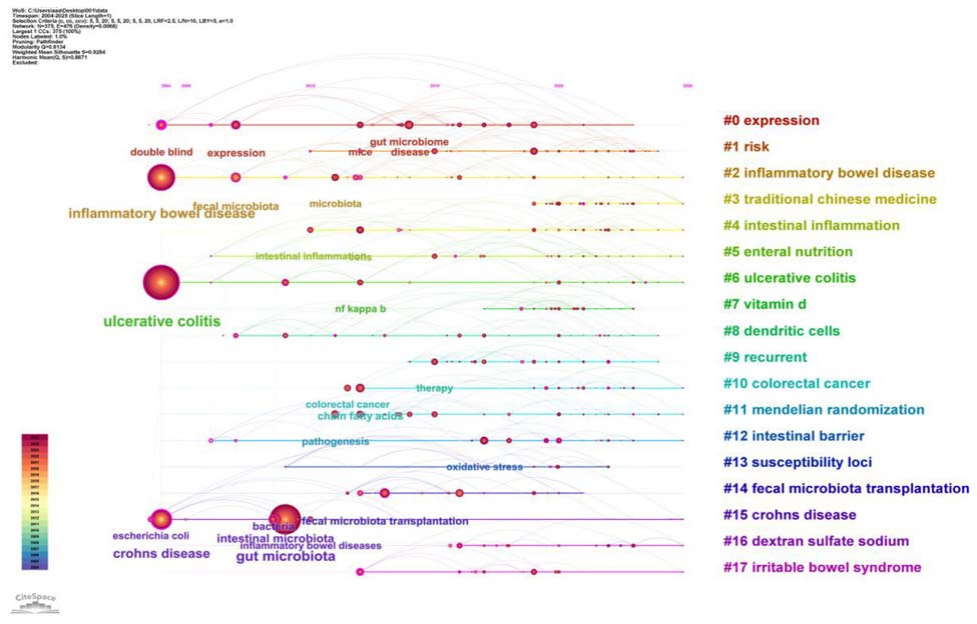
Timeline chart of keyword clustering on gut microbiota and UC. Nodes represent keywords positioned on the timeline according to their year of first appearance, with node size proportional to citation frequency. Node colors transition from blue (older) to red (recent) to indicate temporal progression. Lines between nodes represent co-occurrence or citation links, with thickness representing link strength. The x-axis represents the time span (2004–2025), while the y-axis lists the identified clusters.

The timeline diagram (Figure 13) illustrates the frequency of keyword appearances across different time periods and their evolving trends. Each node in the diagram represents a keyword, with node size indicating frequency of occurrence. Connections between nodes denote co-occurrence relationships among keywords. The timeline reveals research trends from 2004 to 2025. Keywords such as “ulcerative colitis,” “gut microbiota,” and “inflammatory bowel disease” began appearing around 2004 and gradually increased in subsequent years, indicating these were early research focuses in the field. In recent years, keywords like “fecal microbiota transplantation,” “intestinal barrier function,” “intestinal inflammation” and “intestinal epithelial cells” have seen increased frequency, reflecting a trend toward research diversification and deepening. Additionally, the diagram reveals interconnections between keywords-such as the close relationship between “ulcerative colitis” and both “gut microbiota” and “inflammatory bowel disease”-highlighting the mutual influence and cross-disciplinary nature of these topics.

**Figure 14:**
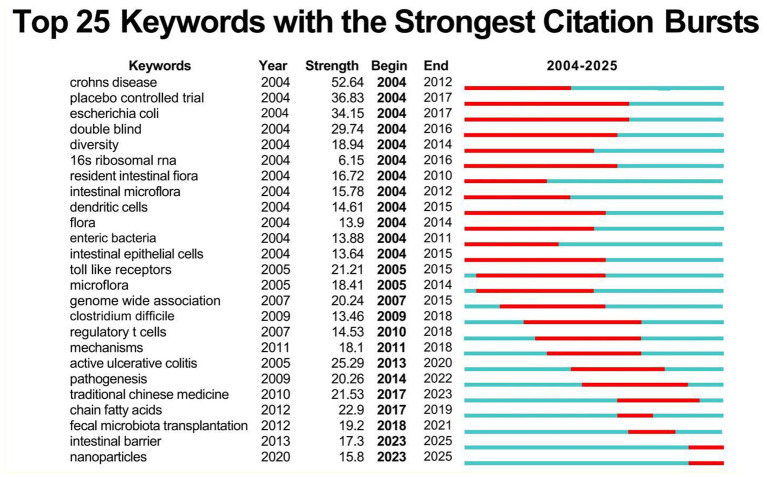
Keyword burst chart of gut microbiota and UC.

Figure 14 details the top 25 keywords in this research field, their burst time ranges, and intensity, with higher values indicating stronger citation outbreaks. Red bars indicate outbreak periods, while blue bars denote non-outbreak periods. During the early outbreak phase (2004–2012), keywords such as “Crohns disease” and “placebo controlled trial” exhibited high outbreak intensity. In the mid-outbreak phase (2013–2016), keywords like “double blind” and “toll-like receptors” became prominent. Recently (2017–2025), “fecal microbiota transplantation/short-chain fatty acids/traditional Chinese medicine” emerged prominently, signaling cross-cultural warming in microbiome interventions. The surge in “fecal microbiota transplantation/traditional Chinese medicine” persisted through 2021/2023, retaining citation potential. The sole entry targeting the “intestinal barrier/nanoparticles” with a 2025 endpoint indicates that nanotechnology-based barrier repair is in the early stages of a citation explosion, positioning it as a highly cited frontier for the next 3 years.

## Discussion

### General distribution

This study conducted a comprehensive visualization analysis of literature on the correlation between gut microbiota and UC within the Web of Science and Dimensions databases using CiteSpace and VOSviewer software. Current research on gut microbiota and UC has formed a multidimensional, interdisciplinary research landscape. Findings indicate that since 2004, literature in this field has shown a fluctuating upward trend, with particularly significant growth from 2016 to 2022. The reason lies in the fact that the notion that “microbes can also cause inflammatory bowel disease” was first proposed in October 2015, challenging the previous fundamental consensus that “gut microbiota can be used to treat inflammatory bowel disease.” Furthermore, it was further proposed that viruses and fungi are also common components of the gut microbiota and may influence the onset and progression of inflammatory bowel disease. Future research requires better characterization of resident viruses in the intestinal, their respective host microorganisms, and how they influence microbial and mammalian physiology. This provides a research direction for subsequent studies in this field (Hansen, 2015). A 2016 review further summarized the efficacy of FMT for treating UC, noting conflicting results and inspiring researchers to refine experimental designs, laying the groundwork for achieving satisfactory therapeutic outcomes in the future (Bamias et al., 2016). Prominent authors in this field include Gasbarrini, Antonio, Chen, Wei, and Cammarota, et al. Among publishing journals, INFLAMMATORY BOWEL DISEASES, NUTRIENTS, and INTERNATIONAL JOURNAL OF MOLECULAR SCIENCES rank at the forefront in terms of publication volume. At the national level, China leads globally with 2,559 publications, followed by the United States with 1,181. Clustering and emergence analysis reveal key research directions: the role of gut microbiota in UC, clinical practice of FMT, and the mechanisms of TCM influencing inflammatory bowel disease via gut microbiota. Co-occurrence maps reveal heightened recent interest in topics like “fecal microbiota transplantation,” “intestinal barrier,” and “intestinal epithelial cells.” Overall, the evolution of gut microbiota-UC research themes shows: the intermediate phase (2013–2016) shifted toward mechanism exploration, including gut microbiota dysbiosis and immune regulation; the recent phase (2017–2025) has seen SCFAs, TCM, FMT, intestinal barrier, and nanoparticles emerge as current research hotspots and frontiers. These findings and trends indicate that research on gut microbiota and UC has evolved from initial clinical observations to mechanism studies, subsequently driving clinical translation based on these findings. Concurrently, research protocols are being refined during translation to enhance clinical efficacy. Concurrently, research in this field has evolved from single-discipline approaches to multidisciplinary collaboration, shifting from a Western medicine-dominated paradigm toward integrated Chinese and Western medicine. Substantial future progress in this area will require more high-quality clinical studies and standardized protocols.

## Frontier topics

**Figure 15:**
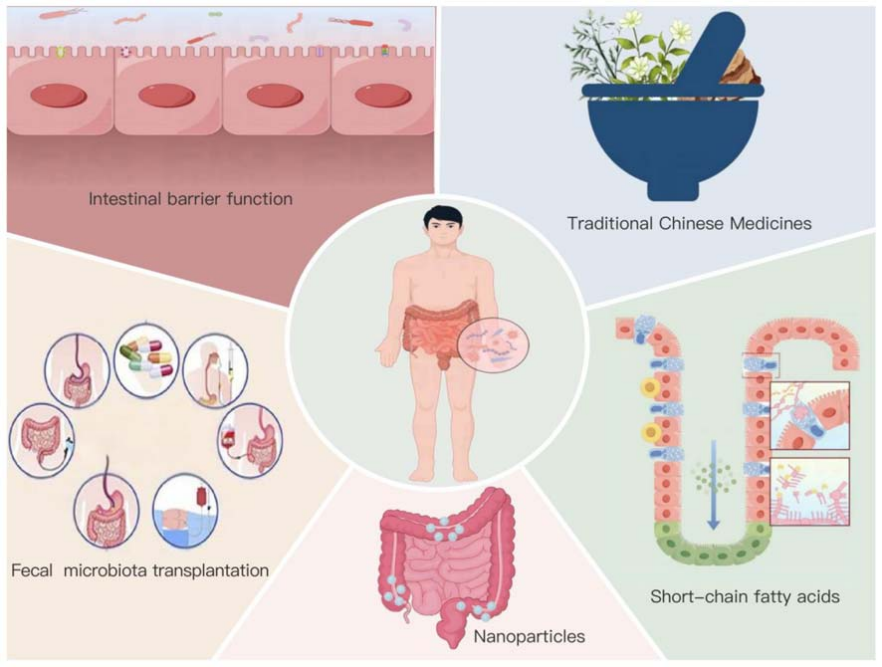
Hotspots and frontiers in research on gut microbiota and UC.

We employed a citation-based evidence hierarchy to identify foundational literature underlying each detected hotspot. For each of the five emergent frontiers identified via burst detection (Figure 15)—intestinal barrier, short-chain fatty acids, traditional Chinese medicine, fecal microbiota transplantation, and nanoparticles—we constructed a core evidence set comprising: (i) the top 10% most-cited papers within each thematic cluster from our dataset (identified via VOSviewer co-citation analysis); (ii) publications by core authors identified via Lotka's law analysis (Table 2) with average citations >100; and (iii) seminal reviews published in Zone 1 journals (Table 3) that specifically addressed the respective mechanisms.

### Mechanism of intestinal barrier damage inducing UC

UC is a chronic idiopathic inflammatory disease confined to the colon and rectum, with compromised intestinal barrier integrity as one of its core pathological features. Recent studies widely recognize that dysbiosis in microbiota-host interactions is a key driver of disease onset and persistence, particularly characterized by dual dysbiosis in microbial composition and metabolic profiles (Schirmer et al., 2019). Metagenomic sequencing reveals a significant reduction in multiple anti-inflammatory or butyrate-producing commensal bacteria within the intestines of UC patients, such as Clostridium clusters IV and XIV species, Bacteroides, Bifidobacteria, Lactobacillus species, Blautia, Faecalibacterium, and Ruminococcus species (Swidsinski et al., 2005; Gophna et al., 2006; Frank et al., 2007; Shan et al., 2021). Conversely, the relative abundance of opportunistic pathogens *Clostridioides difficile* and Enterobacteriaceae species shows compensatory increases (Atarashi et al., 2011). Accompanying these changes in microbial community structure, concentrations of beneficial microbial metabolites—such as SCFAs, secondary bile acids, and indole derivatives—in the intestinal lumen also decreased synchronously (Hu et al., 2022). This further impairs epithelial energy supply, immune regulation, and barrier homeostasis, thereby exacerbating the inflammatory cascade in UC.

Certain genetic alterations inherent to UC can compromise the mechanical and chemical barriers of the intestine. The nuclear receptor Hepatocyte nuclear factor 4 alpha (HNF4α) serves as a key transcriptional regulator for maintaining TJ integrity, directly upregulating the transcription levels of Zonula occludens (ZO)-1, occludin, and claudins. However, HNF4α expression is markedly diminished in intestinal segments of UC patients. Mice with intestinal epithelial cells (IEC)-specific HNF4α knockout exhibit more severe inflammatory responses, further confirming its barrier protective function (Ahn et al., 2008; Cattin et al., 2009). Concurrently, E-cadherin—essential for maintaining intercellular adhesion and epithelial homeostasis—is downregulated. Its encoding gene CDH1 shows markedly reduced expression in UC colonic tissue, impairing lateral adhesion and limiting IEC proliferative potential (van Roy and Berx, 2008). Regarding the chemical barrier, reduced Mucin 2 (MUC2) secretion not only diminishes mucus layer thickness but also alters its glycosylation profile. Abnormally high FUT8 expression in UC patients elevates MUC2 fucosylation levels, disrupting the MUC2/MUC5AC ratio. This leads to loosening of the mucus network structure and increased permeability, creating a “shortcut” for bacterial translocation and amplified inflammation (Yao et al., 2021). This increased permeability exacerbates the disruption of the intestinal mechanical barrier by pathogens and activates the intestinal immune barrier (Cantero-Recasens et al., 2022).

As a chronic inflammatory disease primarily affecting the colon and rectum, the pathogenesis of UC is closely associated with an imbalance in the intestinal immune barrier. During disease activity, the number of M1 macrophages in the patient's intestine significantly increases, and the various pro-inflammatory cytokines they secrete (such as IL-6 and TNF-α) play a key role in amplifying inflammation (Na et al., 2019). These cytokines not only disrupt the integrity of the intestinal epithelial barrier by downregulating TJ protein expression but also induce apoptosis in IECs, thereby exacerbating mucosal damage. Furthermore, IL-6 and TNF-α can activate the NF-κB pathway by activating the STAT3 signaling pathway. Specifically, TNF-α promotes the degradation of IκBα, enhancing the phosphorylation and nuclear translocation of the NF-κB p65 subunit. This leads to sustained activation of downstream inflammation-related gene expression, driving the persistent progression of the inflammatory response (Zhang J. et al., 2023; Zhang M. et al., 2023). Clinical studies demonstrate that TNF-αantagonists like infliximab significantly alleviate clinical symptoms in UC patients by neutralizing TNF-α activity, further validating its central role in UC inflammation (Danese et al., 2022). At the level of immune regulation, macrophages not only activate T cells through antigen presentation but also promote the differentiation and expansion of pro-inflammatory T cell subsets like Th1 and Th17 cells by secreting TNF-α. The synergistic action of IFN-γ secreted by Th1 cells and macrophage-derived TNF-α further induces IEC apoptosis, exacerbating mucosal barrier disruption. Conversely, the proportion of regulatory T cells (Tregs) with immunosuppressive functions is significantly reduced in UC patients, suggesting impaired immune tolerance mechanisms (Duan et al., 2023). Notably, recent studies have identified a substantial number of stem-like T cells within UC inflammatory regions, which may play a crucial role in sustaining the inflammatory state. Further investigations indicate that deletion of key transcription factors B-cell lymphoma 6 protein (BCL)-6 or T cell factor 1 (TCF1) in CD4^+^ T cells substantially reduce colonic T cell populations and impairs their capacity to sustain effector T cell populations, thereby alleviating inflammatory responses. This discovery offers new insights into the functional heterogeneity of T cell subsets in UC.

The above studies integrate the “microbiome-epithelium-immune” tripartite barrier and systematically describe the mechanism by which intestinal barrier damage induces UC through combined evidence from metagenomics, epigenetics, cytokines, and T cell subsets. However, current research predominantly relies on Western populations. Do barrier damage characteristics align in Asian populations with early-onset, elderly, obese, or smoking-related UC? Large-scale cross-ethnic cohorts remain lacking. Existing models rely on “weight loss and histological scores” as gold standards, lacking the endpoint of “barrier repair quality”—even if inflammation subsides, recurrence remains inevitable if the barrier is not fully rebuilt. Furthermore, the direction of causality remains unclear. Is dysbiosis a “trigger” or a “secondary hit”? Antibiotics or FMT only partially alleviate colitis in mice, suggesting microbiota is not the sole driver. Future studies require establishing a multi-center longitudinal cohort across Asia-Africa-Latin America (pre-treatment → induced remission → deep remission → post-pouch surgery) to compare the impact of genetic backgrounds on barrier repair speed. By integrating metagenomics, serum/fecal metabolomics, IEC-specific cfDNA methylation, and intestinal ultrasound contrast parameters, machine learning models can predict high-relapse populations exhibiting “inflammation resolution without barrier repair,” enabling precision maintenance therapy.

### SCFAs can regulate intestinal epithelial barrier function and gut microbiota

SCFAs, as core metabolites of the gut microbiota represented by acetate, propionate, and butyrate, significantly accelerate the repair of the intestinal epithelial barrier. This barrier, composed of columnar epithelial cells and goblet cells, relies on the multi-target regulation of SCFAs for its integrity (Fukuda et al., 2011). Butyrate stimulates cell proliferation and enhances barrier function by inducing intestinal epithelial cells to secrete IL-18 (Singh et al., 2014; Chiang et al., 2022). This effect is primarily mediated by the G protein-coupled receptor GPR109a; GPR109a deficiency exacerbates colitis in mice, while the agonist nicotinic acid alleviates inflammation by activating this receptor, further confirming the protective role of the butyrate-GPR109a axis. Furthermore, (Deleu et al. 2023) utilized an organoid monolayer model derived from UC patients to demonstrate that high concentrations of acetate exhibit low toxicity, upregulate the proliferation marker MKI67, promote epithelial hyperplasia, and significantly increase the expression of barrier-related genes MUC2 and CLDN1. This enhances the integrity of the mucus layer and tight junctions (TJs).

The integrity of the epithelial barrier depends on the integrity of tight junctions (TJs), whose core structure comprises transmembrane components (occludin, claudin, JAM) and cytoplasmic scaffolding ZO proteins (Capaldo et al., 2017; Van Itallie and Anderson, 2017; Tsukita et al., 2019). SCFAs can fine-tune the TJ network through both transcriptional and post-translational mechanisms. (Saleri et al. 2022) observed “molecular fingerprint-like” regulation of TJ proteins by different SCFAs in a porcine intestinal epithelial model: butyrate specifically elevated ZO-1 and occludin levels while having minimal effect on claudin-4; acetic acid significantly induced occludin and claudin-4 without altering ZO-1; lactic acid acted exclusively on ZO-1; only propionic acid simultaneously upregulated all three proteins, suggesting the broadest spectrum of TJ-enhancing effects. Beyond expression levels, SCFAs also participate in TJ spatial reorganization. (Miao et al. 2016) reported that sodium butyrate promotes TJ redistribution in Caco-2 monolayer cells by inhibiting the MLCK/MLC2 pathway and reducing PKCβ2 phosphorylation, thereby restoring barrier function. Collectively, SCFAs remodel TJ architecture through “ligand-protein” specificity, providing precise targets for enhancing intestinal barrier function.

Beyond tight junctions, SCFAs can also upregulate the expression of various barrier-associated molecules such as mucins and immunoglobulins. (Deleu et al. 2023) demonstrated using UC organoid monolayers that acetate significantly increases MUC2 synthesis, suggesting it directly strengthens the mucus layer. (Willemsen 2003) further discovered that butyrate and propionate similarly promote MUC2 production by inducing goblet cells to release prostaglandin E. Although the specific transduction pathways remain incompletely elucidated, (Le Poul et al. 2003) previously reported that acetate, propionate, and butyrate can activate GPR41 and GPR43; the functional weight of these receptors in mucus secretion requires further validation. Regarding acquired immunity, intestinal IgA is crucial for maintaining microbial homeostasis by neutralizing pathogens and supporting commensal bacteria (Huus et al., 2020). (Wu et al. 2017) indicated that intestinal-derived acetate drives IgA production via GPR43 signaling (Brown et al., 2003; Takeuchi et al., 2021) further confirmed that acetate not only elevates IgA levels but also finely regulates its recognition specificity toward commensals like Enterobacteriaceae. However, whether GPR41 and GPR109a participate in the acetate-IgA axis, and the breadth of antimicrobial activity of this IgA, remain to be systematically elucidated.

The intestinal lumen harbors a highly complex and heterogeneous microbial ecosystem encompassing bacteria, fungi, viruses, and other microorganisms across multiple kingdoms (Milani et al., 2017). Its stable configuration serves as the cornerstone of host intestinal homeostasis, while disruption is closely associated with various diseases including IBD (Heimerl et al., 2006). Under physiological conditions, the microbiota and host engage in mutualistic symbiosis: microorganisms ferment complex carbohydrates, releasing SCFAs, which in turn nourish the epithelial barrier, reinforcing its integrity (LeBlanc et al., 2013). Conversely, SCFAs and SCFA-producing bacteria also shape community architecture. Wang et al. reported that the abundance of the butyrate-producing bacterium *Faecalibacterium prausnitzii* positively correlates with the proportion of bifidobacteria and lactobacilli in the feces and mucosa of IBD patients (Wang et al., 2014). Kumari et al. observed a significant reduction in butyrate-producing Streptococcus and *Streptococcus finestis* clusters in UC patient samples (Kumari, 2013), suggesting that the absence of butyrate-producing bacteria may weaken the anti-inflammatory microenvironment. Butyrate and other SCFAs can also induce epithelial secretion of IL-18, a cytokine that drives the expression of antimicrobial peptides (AMPs) such as defensins, calmodulin, and lipocalin-2 (Elinav et al., 2011). AMPs maintain microbiota-host balance by suppressing overgrowth of potential pathogens like Staphylococcus. Given that AMPs are predominantly synthesized by epithelium during inflammation and directly influence IBD progression, both microbiota composition and AMP levels constitute key bidirectional targets for SCFA intervention in IBD (Ho et al., 2012).

The above studies systematically summarize the molecular mechanisms by which SCFAs exert protective effects in UC through regulating the intestinal epithelial barrier and gut microbiota, emphasizing the “ligand-protein” specificity of different SCFA types (acetic acid, propionic acid, butyric acid) at distinct targets. However, most mechanism studies remain confined to *in vitro* or animal models, lacking prospective cohort or randomized controlled trials to validate the long-term efficacy of SCFA interventions in UC patients. Key uncertainties persist regarding the effective concentration range of SCFAs (e.g., what constitutes “high-concentration acetate”?), the optimal time window for action (acute vs. chronic phase), and site specificity (ileum vs. colon). Furthermore, research has primarily focused on bacterial-derived SCFAs, overlooking potential indirect regulation of SCFA metabolism by fungi (e.g., Candida) or phages through cross-feeding or host interactions. Future studies should establish dose-response models linking SCFA concentrations to barrier repair efficacy (particularly comparing low-dose chronic exposure vs. high-dose acute bolus administration). Tracking real-time SCFA concentration changes across distinct colonic segments (particularly the distal colon where lesions are most severe). Furthermore, clarifying whether SCFAs synergistically enhance barrier function with other microbial metabolites (e.g., indole-3-acetic acid from tryptophan metabolism) remains essential.

### TCM regulate gut microbiota to treat UC

In recent years, research on the use of TCM to regulate gut microbiota for treating UC has increased significantly. Specifically, TCM can treat UC by promoting the growth of beneficial bacteria and reducing the number of pathogenic bacteria. Therefore, TCM shows great promise in treating UC by improving gut microbiota. In a dextran sulfate sodium (DSS)-induced UC mouse model, Coix seed, *Polygonum multiflorum*, and *Prunella vulgaris* remodel the gut microbiota, significantly promoting the proliferation of beneficial bacteria such as Bifidobacterium spp. and Lactobacillus spp. while inhibiting the colonization of potential pathogens like Bacillus spp (An et al., 2019; Wang et al., 2019; He et al., 2021). Huangqin Decoction, Baituoweng Decoction, and Lizhong Decoction alleviate microbe imbalance-driven colitis by reducing the abundance of Clostridium difficile, Enterobacteriaceae, and *Escherichia coli* while simultaneously increasing the proportion of protective microbiota (Zou et al., 2020; Hua et al., 2021; Wu et al., 2022). The ShenLing BaiZhu San and Huanglian Jiedu Decoction can upregulate the relative abundance of SCFA-producing strains such as Lactobacillus and Alternaria spp., improving the microbial metabolic profile and thereby reducing pathological damage to the intestinal mucosa (Gu et al., 2021). Furthermore, Da Huang Mu Dan Tang restores Th17/Treg immune homeostasis by fine-tuning gut microbiota composition: It simultaneously suppresses proinflammatory factors such as IL-6 and TNF-α while enhancing TGF-β production, ultimately alleviating histological lesions in experimental colitis and exerting therapeutic effects (Luo S. et al., 2019; Luo Y. et al., 2019).

Although research on treating UC through modulating gut microbiota using TCM has made some progress in recent years, significant limitations remain. These include insufficient depth in mechanism studies, with most research merely describing how TCM herbs alleviate UC symptoms alongside changes in gut microbiota. There is a lack of robust experimental evidence regarding which specific microbial populations TCM herbs regulate, or through which metabolic products or signaling pathways they influence the intestinal immune system. The complex composition of herbal active ingredients and limited understanding of the specific components and their targets for modulating the microbiota make it difficult to conduct dose-response studies. Therefore, future research on herbal medicine-mediated gut microbiota regulation for UC treatment should focus on elucidating how herbal components are converted into bioactive metabolites by intestinal bacteria, and how these metabolites in turn influence microbial structure and function—achieving “drug-microbe interactions.” Exploring the impact of individual gut microbiota on herbal medicine activity will provide a theoretical foundation for precision herbal medicine application. Utilizing high-throughput sequencing and multi-omics analysis, identify “star” microbiota closely associated with UC that can be significantly modulated by Chinese herbal medicine. Develop targeted monomers, compound formulas, or modern formulations to regulate these “star” microbiota, enhancing treatment precision. Simultaneously, we will clarify the functional characteristics of these key microbial communities—such as short-chain fatty acid production, immune regulation, and barrier repair—and correlate them with clinical efficacy and inflammatory markers to establish a microbiota-function-therapeutic effect linkage. This approach will better elucidate the scientific basis of TCM treatment for UC, advancing the modernization and individualized application of TCM.

### The current landscape of FMT in treating UC

The gut microbiota is a complex multicellular community. Approximately 3.3 million microbial genes, up to 10 bacterial phyla, and 1,000 bacterial species (90% of which belong to the Bacteroidetes and Firmicutes phyla) have been identified in the human gut, along with symbiotic fungi and viruses (Macaluso et al., 2024; Sugihara and Kamada, 2024). Compared with the normal control group, the IBD gut microbiota exhibits significant alterations in abundance, diversity, and composition, characterized by reduced anti-inflammatory bacterial abundance and increased pro-inflammatory bacterial abundance, alongside a highly unstable environment (Shan et al., 2021). For example, in the intestines of IBD patients, the abundance of dominant bacterial groups such as Bacteroides and Bacillus significantly decreases, while non-dominant groups like Actinobacteria tend to increase. Additionally, the genus Rhinolophus is significantly reduced in healthy individuals, and individuals with high genetic risk exhibit a higher likelihood of developing IBD (Imhann et al., 2016). FMT is also known as fecal bacterial therapy, human probiotic infusion, fecal transplantation, intestinal microbiome restoration, and fecal transfer. FMT aims to alter the composition and function of the recipient's gut microbiota by collecting stool from pre-screened healthy donors (HD) and delivering the prepared serum to the patient's gastrointestinal tract via nasogastric tube, colonoscopy, or enema (Porcari et al., 2023). FMT has gained widespread popularity in recent years due to its successful treatment of *Clostridioides difficile* infection (CDI), and its application is encouraged in patients with inflammatory bowel disease to restore microbial balance in their intestinal microbiome (Porcari et al., 2023).

Existing animal studies primarily rely on mouse or rat IBD models induced by DSS. The gut microbiota reshapes the immune microenvironment in IBD by participating in both innate and adaptive immune responses (Conrad et al., 2019). In a DSS-induced IBD mouse model, Burrello et al. found that FMT intervention led to decreased levels of proinflammatory genes, antimicrobial peptide genes, mucin genes, interferon-gamma (IFN-γ), interleukin-17 (IL-17), and IL-13, with restoration of the microbial ecosystem (Burrello et al., 2019). In a DSS-induced IBD mouse model, FMT intervention using HD bacteria significantly reduced inflammatory myeloperoxidase (MPO) levels and increased IL-10 expression (Yang et al., 2022). In a 2,4,6-trinitrobenzenesulfonic acid (TNBS)-induced IBD rat model, Qiu et al. demonstrated that FMT suppressed transforming growth factor-β1 (TGF-β1) while upregulating anti-inflammatory Smad proteins, thereby improving disease activity and histology (Qiu et al., 2022). As shown above, FMT can suppress chronic intestinal inflammation by modulating immune function and gut microbiota composition. Additionally, Huang et al. established a mouse model of DSS-induced colitis and administered FMT therapy (Huang et al., 2022). The study found that FMT significantly alleviated colitis symptoms in mice, improved disease activity indices, body weight, and colon length, and effectively restored the pathological structure of colonic tissue. Moreover, FMT partially restored the expression of tight junction protein ZO-1, promoted goblet cell repair, and thereby enhanced intestinal mucus secretion function. DSS-treated mice exhibited a high proportion of IgA/G-targeted bacteria, which returned to near-normal levels after FMT treatment. FMT also significantly reduced the proportion of IgA/IgG+ B cells, suggesting it may restore intestinal immune responses by regulating IgA/IgG+ B cell function. Overall, FMT ameliorated DSS-induced colitis and exerted therapeutic effects by modulating IgA/IgG-mediated immune responses.

Gut microbiota dysbiosis is closely associated with the onset of UC and participates throughout its entire pathogenesis and progression. UC patients exhibit reduced bacterial diversity and decreased abundance of dominant bacterial communities, alongside increased levels of multiple pro-inflammatory bacteria. These bacteria directly or indirectly contribute to inflammation (Nakase et al., 2021). Repeated administration and multi-donor FMT is effective in alleviating or curing active colitis (Shen et al., 2018). In a randomized controlled trial, patients with active UC receiving FMT demonstrated significantly higher remission rates and more diverse microbiota phenotypes compared to the placebo group, with good biosafety (Moayyedi et al., 2015). This study confirms the efficacy of FMT in treating active UC and highlights the need to focus on fecal donors and duration of colitis to achieve optimal outcomes. In a randomized controlled trial conducted across three Australian hospitals, multi-donor enhanced FMT treatment significantly promoted higher remission rates and endoscopic response rates in patients with active colitis compared to the placebo group, with fewer adverse reactions (Costello et al., 2019). Additionally, (Mańkowska-Wierzbicka et al. 2020) found that patients with colitis undergoing multiple FMTs exhibited increased abundance of Lactobacillus, Micrococci, Prevotella, and TM7 phylum. Oral administration of the EW055 clone, as assessed by 16S rRNA sequencing, reduced the families Staphylococcaceae and Bacillaceae. Multiple FMTs facilitated microbial ecosystem restoration and demonstrated superior efficacy in alleviating active UC. In the trial by (Crothers et al. 2020), compared with sham treatment, patients with UC receiving encapsulated oral fecal microbiota transplantation (cFMT) demonstrated higher clinical remission rates, more pronounced gut microbiota changes, increased CRP, and reduced fecal calprotectin, IL-17A, and IFN-γ+MAIT cells. Thus, oral FMT contributes to prolonging the persistence of intestinal bacterial community structural changes and demonstrates superior efficacy in UC. Additionally, dietary intervention may play a crucial role in enhancing the efficacy of FMT for treating UC. (Leibovitzh et al. 2024) compared two approaches using UC-exclusion diet (UCED) combined with FMT for treating UC: one with donor dietary modification and one without. Results showed that patients receiving donor FMT with dietary modification and UCED exhibited significant beneficial microbial changes in the gut, reduced intestinal inflammation, and decreased fecal calprotectin levels. These findings suggest that combining dietary interventions for both donors and patients may enhance the therapeutic efficacy of FMT in treating UC.

**Figure 16:**
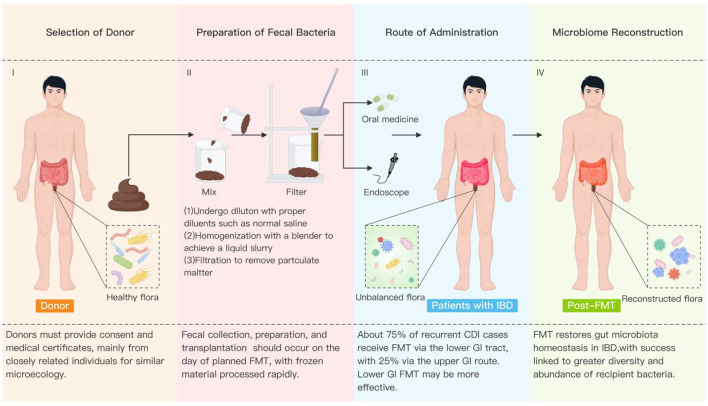
Flow chart of FMT. FMT is an effective technique to achieve intervention and treatment of IBD, which mainly includes donor selection, fecal bacteria preparation and preservation, route of administration, and observation of outcomes related to microbiome reconstruction. FMT involves extracting specific flora from the stool of selected healthy donors through mixing, stirring, and filtering steps, and then transplanting them into the intestine of recipient patients with IBD by oral administration or enema, to rebuild the intestinal micro-ecosystem of the recipient patients with IBD. CDI, *Clostridioides difficile* infection; FMT, fecal microbiota transplantation; GI, gastrointestinal; IBD, inflammatory bowel disease.

However, the application of FMT in IBD treatment faces numerous unresolved challenges, such as standardization of stool preparation, treatment protocols, mechanisms of action, and individual variability. First, regarding safety assessment, genetic differences between donors and recipients pose varying degrees of biological risk when transplanting another person's stool, including viruses and pathogenic bacteria in the donor, host rejection of the transplanted microbiota, and differences in recipient tolerance (Yau et al., 2024). Adverse event monitoring requires a comprehensive system to oversee the transplant process from IBD preparation to administration (Hediyal et al., 2024). Fecal acquisition primarily involves two types: relatives or friends recommended by the patient, or anonymous, unrelated donors (Ianiro et al., 2014). Additionally, multi-donor FMT may further increase gut microbiota diversity (Andary et al., 2024). However, regardless of the source, current research has not identified an optimal FMT donor. Patients with different IBD genotypes and phenotypes, as well as varying disease severity, exhibit differential responses to FMT. Screening and selection to identify responsive patients remain a key focus (Cui et al., 2016). Second, standardized preparation of FMT fecal bacteria still requires consideration of donor selection (related vs. unrelated, optimal donor status), preparation method (fresh, frozen, or freeze-dried; aerobic or anaerobic), and dosage (single vs. multiple doses; Figure 16). It is also noteworthy that most studies on pediatric FMT have used adult fecal donors (Gu et al., 2022; Chen J. et al., 2023; Chen L. A. et al., 2023). Further confirmation of FMT efficacy and safety across different age groups is needed. Rigorous screening of donors and donor feces remains particularly crucial (Lam et al., 2022). Third, a major challenge in FMT for IBD is the difficulty in achieving sustained remission (Jamal et al., 2023). The condition often requires long-term and repeated FMT treatments. Key aspects requiring careful consideration when developing IBD treatment protocols include stool type, optimal dosage, optimal frequency of administration, and delivery routes for FMT, such as enemas, colonoscopic administration, or oral capsules (Kelly et al., 2017). Clinical remission rates for FMT in IBD are moderate, with substantial variability in patient response even among those receiving identical treatment regimens (Ianiro et al., 2022). These studies indicate significant individual differences in the efficacy of FMT for IBD. The absence of adequate predictive targets or diagnostic models makes it challenging to measure the long-term clinical effectiveness of FMT in IBD patients (Liu et al., 2023). Specifically, exploring the potential impact and long-term benefits of FMT for high-risk IBD patients with poor prognosis represents a promising avenue for investigation (Shao et al., 2023). Furthermore, current research on FMT for inflammatory bowel disease remains limited, with its mechanisms not fully elucidated. Existing studies primarily focus on microbial and metabolomic changes in the recipient gut and their relationship to clinical outcomes (Qu et al., 2022; Yuan et al., 2023). However, many other critical factors, such as mucosal immunity, immune-mediated microbiota regulation, and their interactions with host responses, remain understudied in the literature (Hocking et al., 2022). This limitation indicates gaps in our understanding of the full mechanisms underlying FMT therapeutic effects.

### Nanoparticles treat UC by influencing the gut microbiota

The pathogenesis of UC remains uncertain and may be associated with host genetics, environmental factors, immune dysregulation, and the gut microbiota (Kudelka et al., 2020). Pharmacological treatments for UC (aminosalicylates, immunomodulators, corticosteroids, and monoclonal antibodies) provide only temporary relief, accompanied by side effects and drug resistance (Bischoff et al., 2023). Therefore, there is an urgent need to identify better therapeutic options, particularly those targeting inflamed colon with minimal side effects.

**Figure 17:**
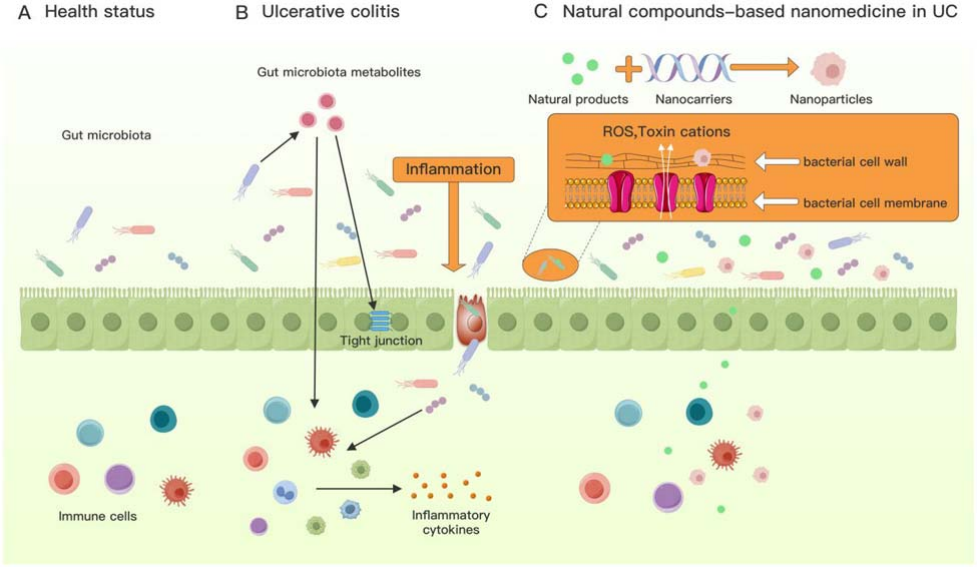
Possible mechanisms of interaction between nanoparticles and the gut microbiota. **(A)** Healthy gut, **(B)** gut with UC, and **(C)** natural compound-based nanomedicine in UC therapy.

Research indicates that nanoparticles can be utilized to restore gut microbiota balance in the treatment of UC (Figure 17). Oral administration of amyloid-polyphenol hydrogels enables prolonged retention within the colon. This hydrogel demonstrated significant positive effects in a mouse model of colitis, improving intestinal barrier function and regulating dysbiosis by reducing colitis-associated bacterial groups, particularly facultative anaerobes such as Aestuariispira and Escherichia. Additionally, short-chain fatty acid metabolites were enriched (Hu et al., 2020). In an acute colitis mouse model, Lee et al. engineered a hyaluronic acid-bilirubin nanomedicine (HABN) that targets inflamed colons and restores the epithelial barrier. HABN significantly increased the abundance of key gut microbiota species crucial for intestinal homeostasis, such as *Akkermansia muciniphila* and Clostridium XIVa, by enhancing overall gut microbiota richness and diversity (Lee et al., 2019). Alfaro-Viquez et al. synthesized hybrid nanoparticles containing cranberry proanthocyanidins-chitin oligosaccharides (PAC-CHTNp) and investigated their effects on the invasion of intestinal epithelial cells by ExPEC. Results showed that PAC-CHTNp significantly inhibited invasion of intestinal epithelial cells, enhanced PAC stability, and promoted molecular adhesion between PAC and ExPEC (Alfaro-Viquez et al., 2018). Glycogens modified with uronic acid and α-sulfate groups can self-assemble into nanoparticles. Nanocarriers can encapsulate ginsenoside Rh2 into Rh2 nanoparticles (Rh2 NPs). Rh2 NPs may improve inflammatory conditions and histological scores in UC mice. They also restore gut microbiota diversity, exerting beneficial effects (Xu et al., 2021). Furthermore, studies indicate that plant-derived NPs serve as effective weapons against specific strains. Zhang et al. found that orally administered gingerol-derivative liposomes (GDLPs) specifically targeted *L. rhamnosus* GG. In colitis mice, GDLPs' micro-RNA mdo-miR7267-3p targeted *L. rhamnosus* GG. Monooxygenase was employed to increase indole-3-carboxaldehyde production and improve intestinal barrier function (Zhang et al., 2016). Tong et al. found that mEVs contain numerous immunologically active proteins and regulate intestinal immunity and microbiota in mice. Oral mEV administration prevented colon shortening and reduced intestinal epithelial damage in a rat UC model. Gut microbiota partially recovered following mEV intervention, suggesting mEVs may modulate intestinal immunity by influencing the intestinal microbiome (Tong et al., 2021).

Meanwhile, the gut microbiota can exert both detrimental and beneficial effects on the metabolism of nanomedicines. The gut microbiota can metabolize various natural bioactive compounds, and this metabolism may lead to reduced absorption and bioavailability of natural bioactive products. Bacterial species such as Bifidobacterium, Lactobacillus, and Eucalyptus can catalyze phenolic metabolism (Shabbir et al., 2021; Zhang J. et al., 2023). *Escherichia coli, E. fergusonii* (ATCC 35469), Bifidobacterium, and Lactobacillus play key roles in curcumin degradation (Zam, 2018). *Bacteroides fragilis, Bacillus subtilis*, and *Clostridium perfringens* are bacterial strains converting quercetin into metabolites (Zhang et al., 2014). These bioactive compounds can be encapsulated in nanocarriers to prevent gut microbiota metabolism and maintain stability. The gut microbiota can biotransform colon-released nanomedicines to promote colitis treatment. The gut microbiota can degrade anthocyanins into chloramphenicol derivatives and benzoic acid (Rosales et al., 2022; Luo Y. et al., 2019). These metabolites stimulate or inhibit the growth of other specific bacteria, thereby further regulating the gut microbiota. Anthocyanin metabolites can promote short-chain fatty acid (SCFA) production, lower intestinal pH, and inhibit pathogen growth (Tian et al., 2018). However, despite progress in understanding nanomaterials' effects on the gut microbiota, the precise mechanisms by which nanomaterials influence the microbiota remain unclear. Further research is needed to evaluate the impact of nanomaterials on the microbiota.

### Nanoparticles as the next-generation precision frontier

Figure 14 reveals the temporal hierarchy of innovation waves in the UC-microbiome field, holding profound implications for future research strategies. The identification of “nanoparticles” as the sole keyword triggering a citation explosion by 2025 stands in stark contrast to the keywords emerging after 2017—FMT, SCFAs, and TCM—signaling a paradigm shift toward precision bioengineering that warrants in-depth analysis.

Unlike the period from 2017 to 2023, which primarily focused on microbial interventions (such as FMT and probiotics) and centered on microbial composition, the emergence of nanoparticle technology in 2025 signifies a field-wide transition toward nanoscale structural and functional regulation. This temporal trajectory reveals three distinct developmental phases: (i) Descriptive Correlation Phase (2004–2016, characterized by surges in “Crohn's disease” and “placebo-controlled trials”); (ii) Component Intervention Phase (2017–2023, dominated by FMT and microbial metabolites); (iii) Precision Engineering Phase (2024–present, spearheaded by nanoparticle delivery systems).

The immediacy of nanoparticle emergence (2025 in the dataset) indicates this represents not merely incremental progress but the frontier of disruptive innovation. Bibliometric trajectory analysis predicts this research cluster will likely absorb and integrate prior cutting-edge achievements—combining microbiological insights derived from fecal microbiota transplantation (FMT) with nanoscale delivery technologies to precisely target intestinal barrier defects. In keyword burst analysis, “fecal microbiota transplantation” frequently co-occurred with “intestinal barrier” and “short-chain fatty acids,” suggesting future research will trend toward hybrid therapies (e.g., nanoparticle-encapsulated short-chain fatty acids or nano-carriers enhancing FMT) rather than single modalities.

## Limitations

Undeniably, this study has several limitations. Initially, our data was sourced solely from the WoSCC database and Dimensions database, which exclusively includes English-language publications in the formats of Articles and Reviews. The article/review-only restriction excludes article types such as conference abstracts and fast reports, causing a time lag in keyword salience detection (failing to capture early signals of emerging hotspots). The English-only restriction may underestimate research contributions from non-Anglophone countries (e.g., Japan, Germany), thereby distorting geographic trends to reflect the English-language publication ecosystem rather than actual global distribution. Nevertheless, given the minimal proportion of non-English articles, the trends identified in our research remain a valuable reference. Second, by relying primarily on WOSCC citation data, we may have underestimated the impact of publications in journals not indexed in WOSCC but present in Dimensions. Furthermore, the “citation lag” phenomenon means recent high-impact publications may appear deceptively under-cited, potentially skewing burst detection analyses toward older, well-established topics rather than truly cutting-edge discoveries. Third, search results may vary due to differences in the scope of databases purchased by various organizations. Fourth, some analytical results may also exhibit slight existence differences due to differences in software versions and analytical methods.

## Conclusion

This study employs bibliometric methods to reveal research hotspots and developmental trends in intestinal microbiota studies related to UC. Research on the correlation between intestinal microbiota and UC represents an evolving and advancing field. It has progressed from initial basic research and clinical trial design, to in-depth exploration of microbial diversity and intestinal barrier function, and further to the application of modern technologies and efficacy assessment. Research on intestinal microbiota and UC has continuously evolved, encompassing studies on intestinal microbiota abundance, FMT, Western pharmaceuticals, Chinese patent medicines, clinical research, mechanism studies, and basic-clinical translational research. Research findings indicate that current hotspots in intestinal microbiota-UC correlation studies focus on intestinal microbiome dynamics, TCM formulations and active components, FMT, immune function, nanoparticle applications, and mechanisms involving anti-inflammatory effects and intestinal barrier improvement. The field of intestinal microbiota-UC correlation research is currently in a phase of tremendous opportunity. By flexibly applying various detection technologies, strengthening international collaboration, broadening research perspectives, continuously innovating methodologies, and deeply exploring the multi-level interaction mechanisms between intestinal microbiota and UC, this field is poised to achieve new breakthroughs. These advancements will provide robust scientific evidence and powerful technical support for enhancing UC treatment standards and improving the quality of life for UC patients.

## Data Availability

The original contributions presented in the study are included in the article/supplementary material, further inquiries can be directed to the corresponding author.
